# An Application of Analytic Wavelet Transform and Convolutional Neural Network for Radar Intrapulse Modulation Recognition

**DOI:** 10.3390/s23041986

**Published:** 2023-02-10

**Authors:** Marta Walenczykowska, Adam Kawalec, Ksawery Krenc

**Affiliations:** Faculty of Mechatronics, Armament, and Aerospace, Military University of Technology, 00-908 Warsaw, Poland

**Keywords:** radar signal recognition, artificial neural network (ANN), continuous wavelet transform (CWT), analytic wavelet transform (AWT), analytic Morse wavelet, intrapulse modulation recognition, feature extraction, phase-coded waveforms

## Abstract

This article analyses the possibility of using the Analytic Wavelet Transform (AWT) and the Convolutional Neural Network (CNN) for the purpose of recognizing the intrapulse modulation of radar signals. Firstly, the possibilities of using AWT by the algorithms of automatic signal recognition are discussed. Then, the research focuses on the influence of the parameters of the generalized Morse wavelet on the classification accuracy. The paper’s novelty is also related to the use of the generalized Morse wavelet (GMW) as a superfamily of analytical wavelets with a Convolutional Neural Network (CNN) as classifier applied for intrapulse recognition purposes. GWT is used to obtain time–frequency images (TFI), and SqueezeNet was chosen as the CNN classifier. The article takes into account selected types of intrapulse modulation, namely linear frequency modulation (LFM) and the following types of phase-coded waveform (PCW): Frank, Barker, P1, P2, and Px. The authors also consider the possibility of using other time–frequency transformations such as Short-Time Fourier Transform(STFT) or Wigner–Ville Distribution (WVD). Finally, authors present the results of the simulation tests carried out in the Matlab environment, taking into account the signal-to-noise ratio (SNR) in the range from −6 to 0 dB.

## 1. Introduction

Nowadays, Electronic Warfare (EW) is an important element of battlefields. Information about the location of hostile emission sources allows for effective mission planning and ensuring the safety of one’s own resources. At the same time, an increasing number of emissions and extensive research on the implementation of new types of radar waveform [1,2] reflect the complexity of the source identification problem and require the use of flexible solutions allowing for adaptation to changing conditions. For this reason, Artificial Intelligence (AI) algorithms, and in particular Machine Learning (ML) methods, which can be trained with new data appearing during the operations, seem to be an inseparable element of a modern EW system. However, in order for the application of ML to be effective, appropriate analyses and research works related to signal processing and the feature extraction process should be carried out. Recently, the use of TFI obtained with STFT, WVD or continuous wavelet transform (CWT) is often considered [3,4,5,6]. The proposal for a radar signal recognition method based on TFI and high-order spectra analysis is presented in [7].

A very important aspect that should be considered when using TFI for signal recognition algorithms is the analysis of the influence of the type, shape, and length of the window, in the cases of STFT or WVD, or the type and parameters of wavelet in the case of CWT, specifically AWT. Transformation parameters, when selected incorrectly, may significantly affect the classification capabilities. For this reason, in this article, the authors presented their simulation studies related to the influence of generalized Morse wavelet (GMW) parameters on the classification accuracy of the proposed method. This allows to obtain comparable or even higher accuracy than when using WVD, which is currently considered very effective in this respect [6]. At the same time, the use of GWT is characterized by much lower computational complexity, resulting in a reduction in time in both the classification process and the network learning. It is worth noticing that all the simulation tests have been carried out using smoothed pseudo-WVD (SPWVD) with a Kaiser window of various shape parameters (α).

The number of algorithms based on TFIs and deep learning proposed in the literature, e.g., [8,9,10] has been dynamically increasing in recent years. However, there is a visible lack of analysis in the field of parameters values selection for proposed time–frequency transforms. The type of applied atom (such as wavelet) or smoothing window used in energy distributions determines the possibility of adjusting to certain characteristics of the signal or the required level of interference reduction. For different parameter values, the same differences are observed in recognition ability for selected waveform types, so it is considered by authors using a set of networks trained with different wavelet types and parameter values and/or adding other methods used for signal recognition purposes and then applying data fusion methods. The problem of intrapulse modulation recognition with a fusion network is presented in [4]. Data fusion techniques are successfully used in areas such as tracking systems [11] or multisensory systems for unnamed aerial vehicles (UAV) detection [12].

An integral part of the EW system is signal intelligence (SIGINT). In the case of communications intelligence (COMINT) systems, the key information is the type and level of the modulation system used by the enemy. Intercepting this information not only enables to identify the source but also facilitates demodulation, decoding, and decryption of the transmitted signal. Typically, the ability to recognize common modulation types such as Phase Shift Keying (PSK), Frequency Shift Keying (FSK), Amplitude Shift Keying (ASK) or Quadrature Amplitude Modulation (QAM) is considered [13,14]. There are also studies taking into account the possibility of detection of OFDM transmission, among other communication signals [15,16]. In the case of electronic intelligence (ELINT), the main task of the system is to detect, classify, and determine the location of the emission sources other than communication. Therefore, these are particularly radars. The structure and parameters of the radar signals depend primarily on their intended use, no matter if they are early warning radars, short-, medium-, and long-range missile systems radars, as well as aircraft radars, jamming systems, or any other. Typical radar waveforms considered in most studies are continuous wave (CW) or pulses with LFM, stepped frequency modulation (SFM), and phase coding (PC) or traditional pulses with no intrapulse modulation applied. However, more complex types of waveform such as those with nonlinear frequency modulation or mixed signals are increasingly being considered [2,16,17].

In ELINT systems, many emission source parameters, such as radio frequency (RF), direction of arrival (DOA), time of arrival (TOA), pulse width (PW), pulse repetition frequency (PRF), intrapulse modulation, etc., are determined. These parameters are stored in the database and constitute the basis of the process of recognizing the emissions detected by the EW system. In order to determine the basic parameters of radar signals, it is necessary to perform signal processing and the selection of features allowing for effective classification. This issue is a dynamically developing area of research [18,19,20,21,22], while measuring the parameters of specific radar signals in real time still remains a challenge.

The radar emission identification process is usually carried out using a knowledge-based approach. In [18], methods for determining specific radar signal parameters (signatures) are discussed. In [20], the authors analyze the role of estimation accuracy of the arrival time of each step of the pulse. As it turns out, it may affect the determination correctness of such parameters as PW and PRF. Moreover, the application of the wavelet transform (WT) as well as Haar wavelet as tools for sorting radar signals has also been proposed. Compared with the traditional pulse repetition interval algorithm based on a statistics histogram, the method based on WT is characterized by the high accuracy of the arrival time. In [19], three characteristic parameters, namely CWT eigenvalue, frequency domain moment kurtosis coefficient, and frequency domain moment skewness coefficient, have been used in order to recognize the radar signal. The following signals have been considered: polynomial phase signal, pseudocode phase modulation and sinusoidal frequency modulation, product composite of pseudocode phase modulation and LFM, and convolution composite of pseudocode phase modulation and LFM. For a signal-to-noise ratio (SNR) higher then 0 dB, the probability of signal recognition is claimed to be greater then 98%.

The aim of this article is to present an algorithm for automatic intrapulse modulation recognition with use of an AWT and CNN. The proposed solution is based on the use of generalized Morse wavelet (GMW) as a superfamily of analytic wavelets. The properties of the GMW are described in Section 2.3. A significant advantage of using CWT for signal recognition purposes is the possibility of using CWT coefficients at individual stages of signal analysis, e.g., to estimate selected parameters of a radar signal. The CWT is successfully used in algorithms for sorting radar pulses and determining parameters such as pulse repetition time (PRT), pulse width (PW) or time of arrival (TOA) [20].

The research presented in the paper brings a new contribution to the area of radar signal recognition techniques with the use of TFI. In particular, the authors consider:

- Using AWT (with GMW applied) for radar intrapulse recognition purposes instead of popular WVD, SPWVD or STFT;
- Performance metrics comparing methods with AWT and SPWVD used as TFI;
- The influence of GMW parameters values on classification accuracy;
- Applying SqueezeNet as a CNN classifier.

A significant part of the conducted simulation works has been focused on the study of the influence of generalized Morse wavelet parameters on the properties of the analyzed method. The simulation results are presented in Section 3. The usefulness of AWT is mainly due to the ability to observe the instantaneous amplitude, phase, and frequency of signals simultaneously. The appropriate selection of the wavelet parameters allows, in turn, to obtain the required resolution in frequency and to emphasize specific features of the analyzed signals. The simulations, necessary to test the proposed method and confirm its potential effectiveness, were carried out in the Matlab environment.

The use of TFI enables the simultaneous observation of signal features corresponding to the nature of changes in amplitude, frequency or phase over time. This allows for the complete or partial replacement of traditionally used features, calculated usually with the use of FFT, higher-order statistics, instantaneous amplitude, frequency and phase parameters, cepstral analysis, phase constellation, frequency histogram, etc. Moreover, the algorithms of time–frequency signal decompositions with lower computational complexity and lower memory requirements are presented in the literature [23,24]. The computational complexity of time–frequency distributions is presented in [25]. CNNs, on the other hand, are successfully used in image recognition. This is the main motivation for authors to use one of them as the classifier. According to the authors’ knowledge, at present, there are CNN implementations with a significantly reduced structure. An example of such a CNN is SqueezeNet, the implementation of which is available in the Matlab environment. The primary advantages of SqueezeNet, according to [26], are, e.g.,:

- More efficient distributed training;
- Feasible FPGA and embedded deployment;
- Less overhead when exporting new models to clients.

The SqueezeNet structure implementation in Matlab is 18 layers deep. The network has an image input size of 227 by 227, so TFIs rescaling must be applied. A comparison of classification accuracy using TFI with other CNN-type structures is presented in [8].

## 2. Materials and Methods

The number of studies related to the possibility of using ML for signal recognition for electronic warfare and, e.g., cognitive radio, has significantly increased in recent years. When analyzing the literature on signal analysis for ELINT systems, one can see the division of the proposed algorithms into two areas of application. The first one focuses on the analysis of signals, with their duration covering a certain number of symbols or a sequence of radar pulses, and determining their characteristic parameters such as PRI, PW, TOA, etc. [18,20,21,22,27,28]. The second concerns the problem of recognizing intrapulse modulation, which is another important parameter characterizing the [5,29,30] emission source. In both cases, the usefulness of using AI algorithms is emphasized.

There are propositions of algorithms using CWT and ML for recognizing modulations of typical signals used in communications such as ASK, PSK, FSK or QAM [13,14,31,32,33,34]. Moreover, in the field of ELINT, more and more papers are published related to the application of AI for the purpose of recognizing selected types of radar waveforms [3,6,9,10,28,29,35].

Of particular interest is the application of CNNs, where TFIs obtained using STFT, WVD or CWT are fed to the network input. In this context, an important problem that requires special attention is the appropriate selection of the type of transformation and its parameters, so that the input data in the form of TFIs contain features that ensure the required classification accuracy of selected signals. The following subsections present issues related to the usefulness of using CWT to generate TFI and CNN as an classifier of selected pulsed radar signals, with particular emphasis on the influence of the parameters of the generalized Morse wavelet on the classification accuracy.

### 2.1. Radar Waveforms

Applying the pulse compression technology has resulted in a significant increase in the number of signals used in radar technology. Currently, there is a noticeable increase in the number of research works devoted to specific forms of radar signals [1,2], i.e., more and more complex types of radar waveforms ensuring the best possible resolution in time (range) and Doppler frequency (velocity), as well as a high compression ratio. This involves, among other things, the analysis of the ambiguity function, which is also a very useful way of analyzing signal properties in the time–frequency space.

**Figure 1:**
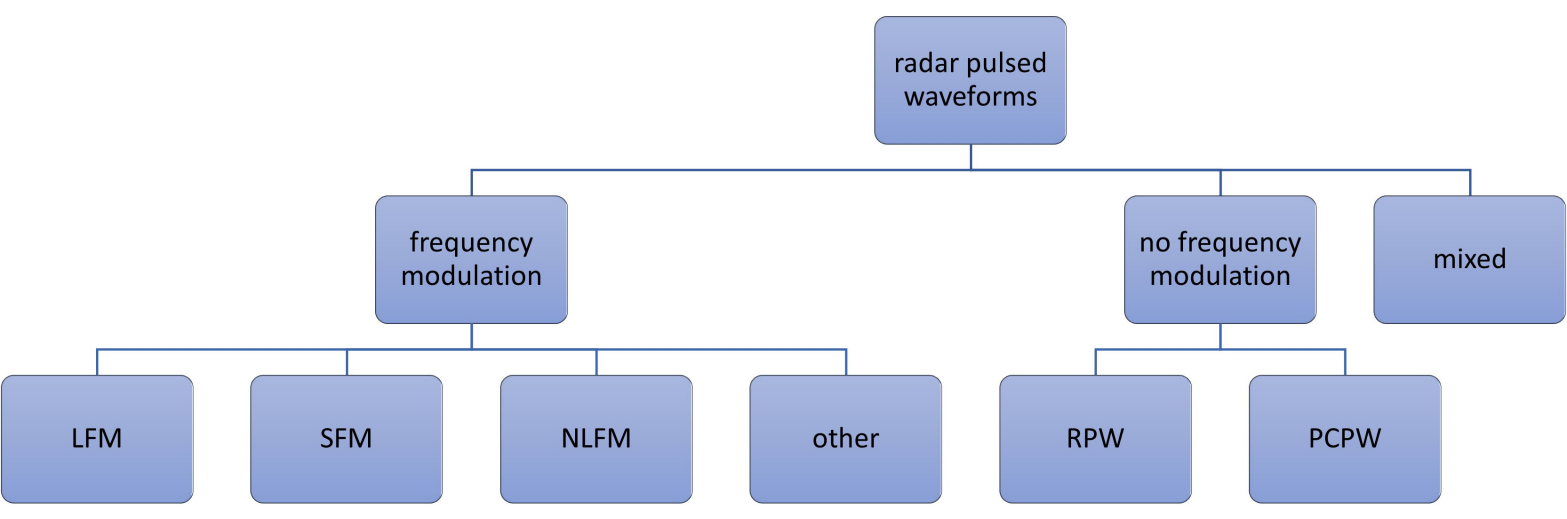
General division of radar waveforms.

The general division of radar waveforms most often taken into account in the research area for automatic recognition of radar signals is presented in Figure 1. Traditional pulsed radar waveforms include rectangular pulses (RP) with continuous wave (CW), LFM, and phase-coded waveforms (PCW). Other types of often considered radar waveforms are NLFM and SFM.

To an NLFM group of signals, authors also assigned quadratic frequency modulation (QFM), sinusoidal frequency modulation (SiFM), etc. There are also works [15] considering the application of OFDM signals for radar purposes. In [2], there is an algorithm for identifying so-called exotic modulations, which include signals modeled as a combination of LFM and Biphase modulation (BPM). In [30], there is also recognition of the same hybrid waveforms as LFM-BPSK and FSK-BPSK. An analysis of combined signals is also presented in [1], in which authors took into account a group called “mixed”, which includes hybrid modulation types (Figure 1). All listed waveforms can be considered in continuous or pulsed form.

For the purpose of this article, the following types of signals were taken into account for simulation: LFM, RP, and PC waveform. In the studies carried out so far, presented in [31], a significant problem was distinguishing between the different types of signals with phase modulation (PM). In most of the presented research results, the classification accuracy in this group of signals is much worse for both COMINT (M-ary PSK and M-QAM signals) [36] and ELINT (PCW) [37]. In order to emphasize the effectiveness of the proposed method, the following types of codes were included in the phase-coded waveform (PCW) group: Barker, Frank, P1, P2, and Px. According to [38], the complex envelope of the PC pulse, with duration *T* and *M* bits in the pulse, is given by equation:

(1) $$x\left(t\right)=\frac{1}{\sqrt{T}}\sum_{m=1}^{M}x_{m}rect\left[\frac{t-\left(m-1\right)t_{b}}{t_{b}}\right]$$

where *M* is called “chip”, $t_{b}=\frac{T}{M}$ is chip number, $x_{m}=exp\left(j\phi_{m}\right)$, the set of *M* phases $\left[\phi_{1},\phi_{2},\ldots ,\phi_{M}\right]$ is the phase code associated with x(t), and rect() is a rectangular function. The first family of phase codes taken into account in our research is Barker. There were *N* = 13 lengths of code chosen for simulations, witch ensures the lowest level of ambiguity function side-lobes ($\frac{1}{N}$) in this code family. There are no longer Barker codes found [39]. The choice of polyphase codes used for simulations (Frank, P1, P2, and Px) is motivated by the fact that difficulties in separating them were expected. The P1, P2, and Px codes are derived from the Frank code [38,39], and they are considered by authors as one family.

The elements of the polyphase codes can be complex depending on the value $\phi_{m}$. For Frank polyphase codes, witch have a length that is perfect square ($M=L^{2}$, where *L* is integer), the definition of its elements $s_{m}\left(1\leq m\leq M\right)$ according to [37,38] is

(2) $$s_{(n-1)L+k}=exp\left(j\phi_{n,k}\right)$$

where 1≤n≤L, 1≤k≤L and:

(3) $$\phi_{n,k}=2\pi \left(n-1\right)\left(k-1\right)/L$$

According to Equation (2), for L=4, we have phase values:

$\left[0\;0\;0\;0\;0\;\frac{\pi }{2}\;\pi \;\frac{3\pi }{2}\;0\;\pi \;0\;\pi \;0\;\frac{3\pi }{2}\;\pi \;\frac{\pi }{2}\right]$

The Frank, *P*1, *P*2, and *Px* codes are applicable only for perfect square length ($M=L^{2}$). The phase of *P*1, *P*2, and *Px* is given by [37,38]:

(4) $$P1:\;\phi_{n,k}=\frac{2\pi }{L}\left[\left(L+1\right)/2-n\right]\left[\left(n-1\right)L+\left(k-1\right)\right]$$

(5) $$P2:\;\phi_{n,k}=\frac{2\pi }{L}\left[\left(L+1\right)/2-k\right]\left[\left(L+1\right)2-n\right]$$

only for even *L*, and:

(6) $$Px:\;\phi_{n,k}=\left\{\begin{array}{c} \frac{2\pi }{L}\left[\left(L+1\right)/2-k\right]\left[\left(L+1\right)2-n\right],\;L\;even\\ \frac{2\pi }{L}\left[L/2-k\right]\left[\left(L+1\right)2-n\right],\;L\;odd \end{array}\right.$$

It should be noted that in the case of the $P_{x}$ code for even *L*, $\phi_{n,k}$ takes the values as for the *P*2 code. For this reason, the simulation parameters should be carefully selected to avoid problems with distinguishing the above codes. For the same reason, the classification was carried out only taking into account the $P_{x}$ code for odd *L*. It was show in [37], where for radar waveform recognition purposes Choi–Williams Distribution (CWD) was considered, that P1 and P2 codes get confused with each other. A similar problem was observed in studies where individual levels of PSK were classified for the communication system recognition algorithm [31]. Similarly, it is expected that, in noisy conditions, the differences in this group of phase codes can be blurred.

### 2.2. The Time–Frequency Transforms in Signal Analysis

Time–frequency (or decomposition) transforms are a very convenient way to present features of different types of waveforms simultaneously in time and frequency domains. The increase in interest in CNNs, particularly observable in the recent years, has made TFIs more and more frequently used schemes in solving the problem of signal classification [3,6]. However, not enough attention is always paid to the influence of the chosen transform parameters on the possibility of enhancing the characteristic features of the signals. This, in turn, may lead to the loss of valuable information and, as a consequence, affect the classification process efficiency. Similarly to the classic approach, the stage of feature extraction determines the effectiveness of the recognition algorithm.

Some of the most popular transforms such as STFT, WVD, and CWT are presented in [40,41]. We can divide them in two classes of solutions: atomic decompositions and energy distributions. The first class includes STFT and CWT, while the second one includes WVD, pseudo-WVD, and SPWVD.

STFT according to [42] is defined as

(7) $$STFT_{x}\left(t,v\right)=\int_{-\infty }^{\infty }x\left(u\right)h^{*}\left(u-t\right)e^{-j2\pi vu}du$$

where h(t) is a short time analysis window localized around t=0 and v=0. The multiplication by the relatively short window $h^{*}\left(u-t\right)$ effectively suppresses the signal outside a neighborhood of time point u=t, so the STFT is a “local” spectrum of the signal x(u) around *t*. The time resolution of the STFT is proportional to the effective duration of the analysis window *h*. In turn, the resolution of the STFT in the frequency is proportional to the effective bandwidth of the analysis window *h*. According to the above, using the STFT cannot achieve good resolution in time and frequency simultaneously. A good time resolution requires a short window h(t), and a good frequency resolution requires a narrow-band filter, and so a long window h(t).

The CWT of a signal x(t) is defined as [42,43]

(8) $$CWT\left(t,a\right)=\int_{-\infty }^{\infty }x\left(u\right)\psi_{t,a}^{*}\left(u\right)du=\frac{1}{\sqrt{\left|a\right|}}\int_{-\infty }^{\infty }x\left(u\right)\psi^{*}\left(\frac{u-t}{a}\right)du$$

where the ψ(u) is called mother wavelet and *a* is the scaling constant. $\psi_{t,a}^{*}$ is called baby wavelet and is the translated and scaled version of ψ(u).

CWT projects a signal x(t) on a family of zero-mean functions, called the wavelets, which are translated and dilated versions of the elementary function, called the mother wavelet. When the scale factor is being modified, the duration and the bandwidth of the wavelet are both changed, but its shape remains the same. In contrast to the STFT, which uses a single analysis window, the CWT uses short windows at high frequencies and long windows at low frequencies. This partially overcomes the resolution limitation of the STFT. STFT and CWT are linear transforms of the signal. Another way of signal analysis consists in distributing the energy of the signal along the two variables of time and frequency. This leads to energy time–frequency distributions, which are naturally quadratic transforms of time and frequency. For STFT, we have a spectral energy density of the locally windowed signal x(u)h*(u−t), defined as [42,43]

(9) $$S_{x}\left(t,v\right)={\left|STFT_{x}\left(t,v\right)\right|}^{2}$$

According to Equation (5) *spectrogram* is a real-valued and non-negative distribution and satisfies the global energy distribution property:

(10) $$\int_{-\infty }^{\infty }\int_{-\infty }^{\infty }S_{x}\left(t,v\right)dtdv=E_{x}$$

In the *CWT* case, the scalogram can be defined [44]:

(11) $$T_{x}\left(t,a\right)={\left|CWT(t,a)\right|}^{2}$$

*CWT* also preserves energy:

(12) $$\int_{-\infty }^{\infty }\int_{-\infty }^{\infty }T_{x}\left(t,a\right)dt\frac{da}{a^{2}}=E_{x}$$

**Figure 2:**
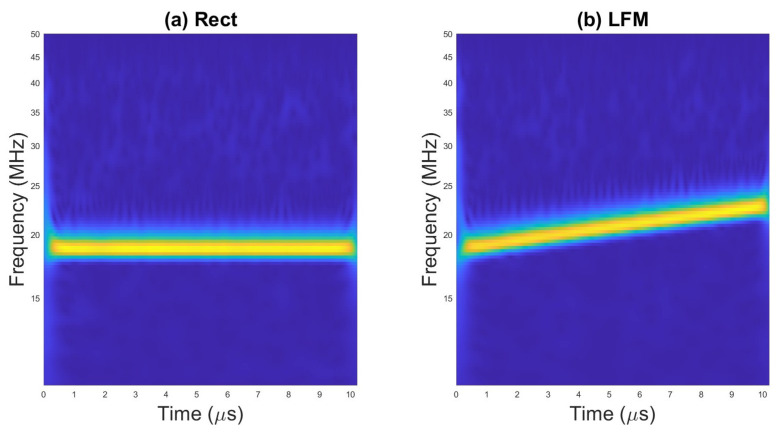
Scalogram for (**a**) rectangular pulse and (**b**) LFM.

**Figure 3:**
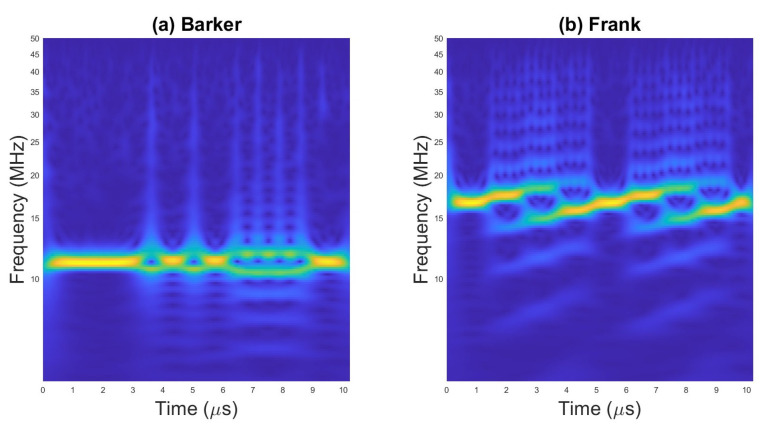
Scalogram for (**a**) pulses with Barker code and (**b**) pulses with Frank code.

**Figure 4:**
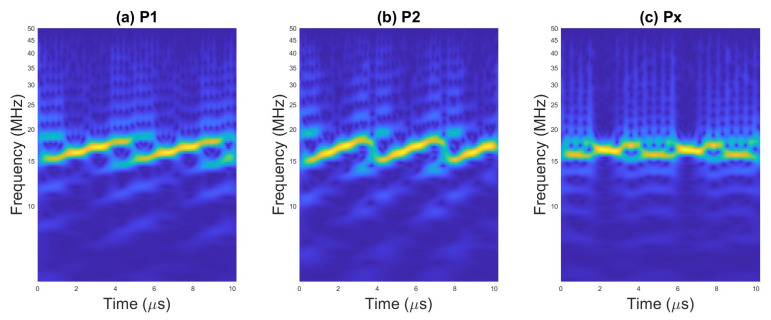
Scalogram for pulses with (**a**) P1 code, (**b**) P2 code, and (**c**) $P_{x}$ code.

An example of scalograms for selected waveforms are presented on Figure 2, Figure 3 and Figure 4.

The second class of time–frequency representations is energy distributions. Some of the most commonly used energy distributions for signal recognition are WVD and CWD. The WVD is defined as [42]

(13) $$WV\left(t,v\right)=\int_{-\infty }^{\infty }x\left(t+\frac{\tau }{2}\right)x^{*}\left(t-\frac{\tau }{2}\right)e^{-jv\tau }d\tau$$

and is a nonlinear decomposition, so the spectrum of two combined signals is not the sum of their spectra but includes so-called cross-spectrum:

(14) $$WV_{x+y}\left(t,v\right)=WV_{x}\left(t,v\right)+WV_{y}\left(t,v\right)+2ℜWV_{x,y}\left(t,v\right)$$

where:

(15) $$WV_{x,y}\left(t,v\right)=\int_{-\infty }^{\infty }x\left(t+\frac{\tau }{2}\right)y^{*}\left(t-\frac{\tau }{2}\right)e^{-jv\tau }d\tau$$

The WVD interference terms will be nonzero regardless of the time–frequency distance between the two signal terms [42]. This could be troublesome and make it difficult to visually interpret the WVD image. A common way to reduce interferences is to use anew distribution, called *pseudo-WV*, defined as:

(16) $$PWV\left(t,v\right)=\int_{-\infty }^{\infty }h\left(\tau \right)x\left(t+\frac{\tau }{2}\right)x^{*}\left(t-\frac{\tau }{2}\right)e^{-jv\tau }d\tau$$

where h(t) is regular window. The windowing operation is equivalent to frequency smoothing. Another modification of WVD is the smoothed pseudo-Wigner–Ville Distribution (SPWVD), defined as

(17) $$SPWV\left(t,v\right)=\int_{-\infty }^{\infty }h\left(\tau \right)\int_{-\infty }^{\infty }g\left(s-t\right)x\left(s+\frac{\tau }{2}\right)x^{*}\left(s-\frac{\tau }{2}\right)ds\;e^{-jv\tau }d\tau$$

According to [42], the compromise of the spectrogram between time and frequency resolutions is in case of SPWVD being replaced by a compromise between the joint time–frequency resolution and the level of the interference terms. Stronger smoothing in time and/or frequency runs into poorer resolution in time and/or frequency.

**Figure 5:**
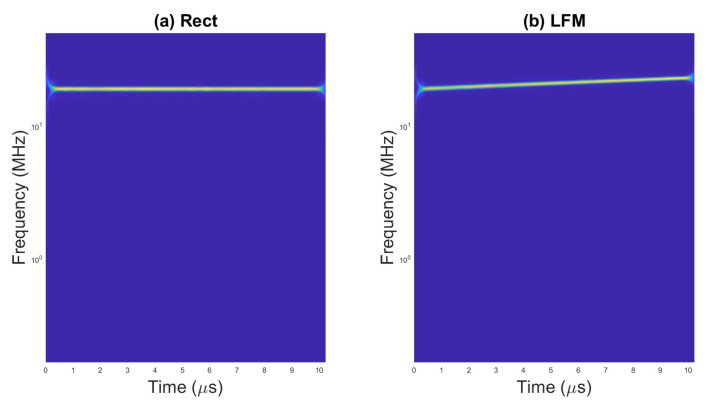
WVD for (**a**) rectangular pulse; (**b**) LFM.

**Figure 6:**
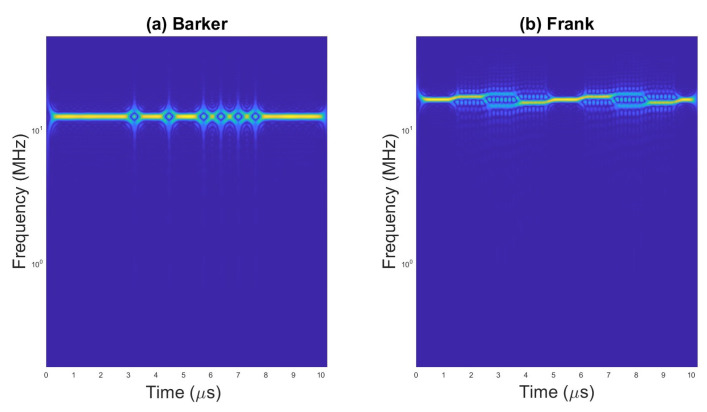
WVD for (**a**) pulse with Barker code; (**b**) pulses with Frank code.

**Figure 7:**
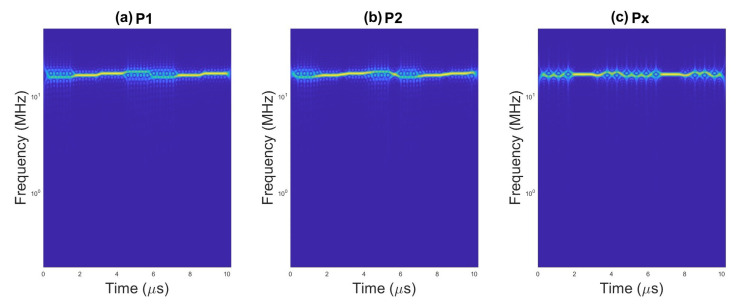
WVD for pulses with (**a**) P1 code, (**b**) P2 code, and (**c**) $P_{x}$ code.

The examples of TFI images for selected radar waveforms obtained with WVD are presented in Figure 5, Figure 6 and Figure 7.

### 2.3. The Analytic Wavelet Transform (AWT) and Generalized Morse Wavelet (GMW)

The continuous wavelet transform (CWT) enables extraction of transient information associated with amplitude/frequency changes and phase shifts, which are characteristics of modulated signals. According to [45,46], it is generally the most useful to describe oscillatory signals and time-localized events in noisy environment, where 1/*s* normalization is more appropriate. Taking the above into account, the definition of the CWT of a signal x(t) takes the form:

(18) $$CWT\left(\tau ,a\right)=\frac{1}{a}\int_{-\infty }^{\infty }x\left(t\right)\psi^{*}\left(\frac{t-\tau }{a}\right)dt$$

where the ψ(t) is called mother wavelet and *a* is the scaling constant. $\psi_{a}^{*}$ is called baby wavelet and is the translated and scaled version of ψ(t). In this case, rescaling time in the input signal as $x(\frac{t}{\rho })$ rescales both the time and the scale of CWT but without changing its magnitude. For analytic wavelets considered in [45,46,47], we have ψ(ω)=0 for ω<0, which means that wavelets vanish for negative frequencies. The AWT is represented in the frequency domain as

(19) $$CWT\left(\tau ,a\right)=\frac{1}{2\pi }\int_{0}^{\infty }\Psi^{*}\left(a\omega \right)X\left(\omega \right)e^{j\omega \tau }d\omega$$

For the analytic wavelet Ψ(ω), maximum amplitude occurs in the frequency domain at $\omega =\omega_{\psi }$, which is called the *peak frequency*. In the case of using an analytic wavelet, such as GMW, there is AWT term used. According to [45], if the value of the wavelet in the peak frequency is set to $\Psi (\omega_{\psi })=2$, then for signal $x\left(t\right)=a_{0}cos\left(\omega t\right)$, we obtain result $|W_{\psi }\left(t,\omega_{\psi }/\omega_{0}\right)\left|=\right|a_{0}|$.

During the simulation studies, the generalized Morse wavelet was taken into account. According to [45,47,48], the Morse wavelet is defined as follows:

(20) $$\psi_{\beta ,\gamma }\left(\omega \right)=U\left(\omega \right)a_{\beta ,\gamma }\omega^{\beta }e^{-\omega^{\gamma }}$$

where $a_{\beta ,\gamma }$ is a normalization constant, U(ω) is the unit step function, and β and γ are parameters controlling the wavelet form. The normalization constant is defined in [47] as

(21) $$a_{\beta ,\gamma }\equiv 2{\left(\frac{e\gamma }{\beta }\right)}^{\frac{\beta }{\gamma }}$$

It follows from the analysis presented in [48] that, by varying the β and γ parameters, the generalized Morse wavelets can take a wide variety of forms. For example, the γ=1 family corresponds to the Cauchy (or Paul) wavelet, the γ=2 correspond to analytic Derivative of Gaussian wavelets, and γ=3 corresponds to Airy wavelets family [47].

**Figure 8:**
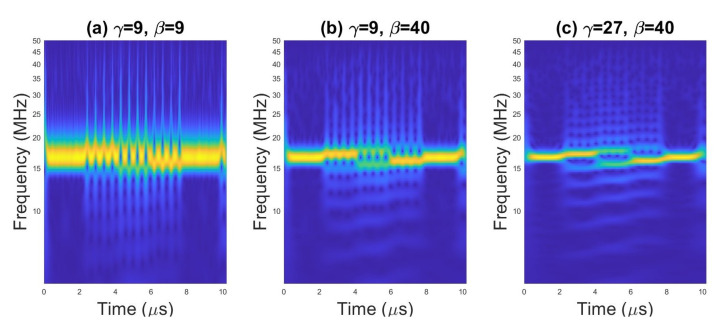
Scalogram for the pulse with Frank code for SNR = 20 dB.

**Figure 9:**
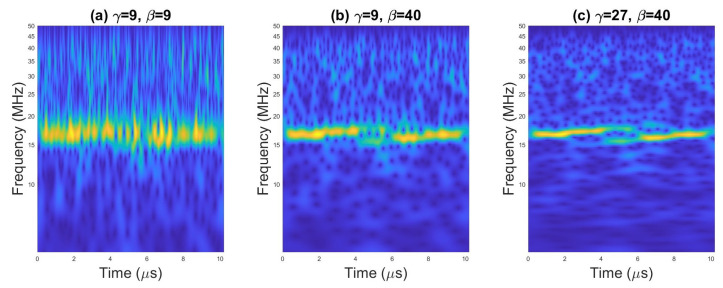
Scalogram for the pulse with Frank code for SNR = 0 dB.

On Figure 8 and Figure 9 are presented the TFIs of radar signal with Frank code (*N* = 16), obtained with AWT and Morse wavelet for different values of γ and β and two values for SNR. There is a significant impact of the parameters of the Morse wavelet on the form of CWT and on the way in which TFI is affected by the increased level of noise. On this basis, it can be assumed that a higher accuracy of the classification can be obtained for selected parameters γ and β. The simulation results described in the following subsections confirm the above statement.

In Matlab Wavelet Toolbox parameters for parameterized analytic Morse wavelet are defined as γ and the time–bandwidth product $P^{2}=\beta \gamma$, and they correspond to the analysis in [45,47,48].

## 3. Results

In order to carry out the research works, the following equipment (hardware and software) was used:

- Dell Precision 3561, i7-1180H, 32GB RAM, NVidia T600, Win11, manufacturer: Dell, Warsaw, Poland;
- MATLAB Version: 9.12.0.2039608 (R2022a) Update 5 with Toolboxes, manufacturer: MathWorks, Inc., Natick, MA, USA.

The simulations were performed for recognition between continuous wave (CW), LFM, and phase-coded waveforms. For signals with phase coding, the same number of chips in one pulse was selected for the *Frank*, *P*1, *P*2 code. Due to the fact that in the *Px* code for even values of *L* the phase $\phi_{n,k}$ takes the same values as in *P*2 code, the *L* was set to the odd one. The simulation parameters of phase-coded waveforms are listed in Table 1.

The conducted research was based on AWT and the use of the Morse wavelet. The authors focused on the analysis of the impact of the wavelet parameters on the obtained classification accuracy. The general simulation parameters are presented in Table 2.

**Figure A1:**
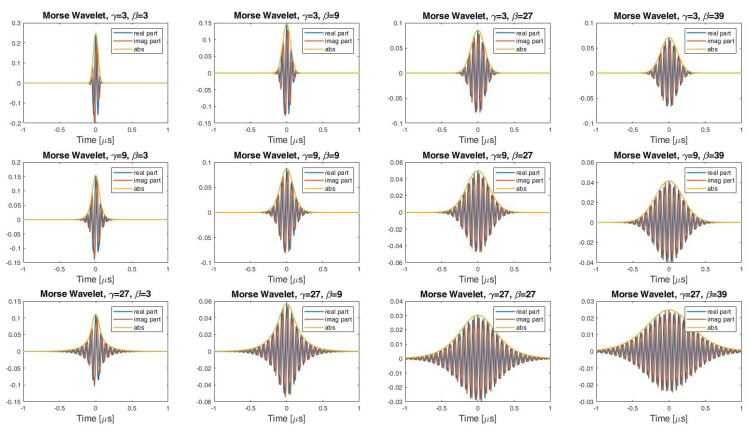
Morse wavelets corresponding to parameters from Table 3 for selected wavelet center frequency.

The simulation tests were carried out taking into account selected values of the γ parameter, and for each of them, the β parameter was changed in the loop. Two parameters per set were considered. The first one was with the analogy with Morse wavelet parameters presented in [45,47,48] and listed in Table 3. Additionally, β=40 was applied. The view of the wavelets corresponding to the parameters γ and β from Table 3 is shown in the Figure A1 in Appendix A.

The second wavelet parameters set is presented in Table 4. The number of oscillations and duration of wavelets was increased (according to change in γ and β) to find out if they could better match the recognized waveform. However, there was no significant increase in classification accuracy noticed.

Only for γ=50 and β=37, the same small increase in classification accuracy was observed. Lower classification correctness also occurred for the same types of PCW: P1 and P2 codes. Generally, classification accuracy remained at the level of about 85–95% (Table 5).

In spite of the lack of significant improvement, it is worth noticing that frequency resolution is much better for higher wavelet parameters values. This can be useful for frequency-modulated waveform recognition and methods for optimal scale selection for emitter parameters estimation e.g., pulse duration (PD), sweep frequency, pulse repetition interval (PRI), time of arrival (TOA), and others.

**Figure 10:**
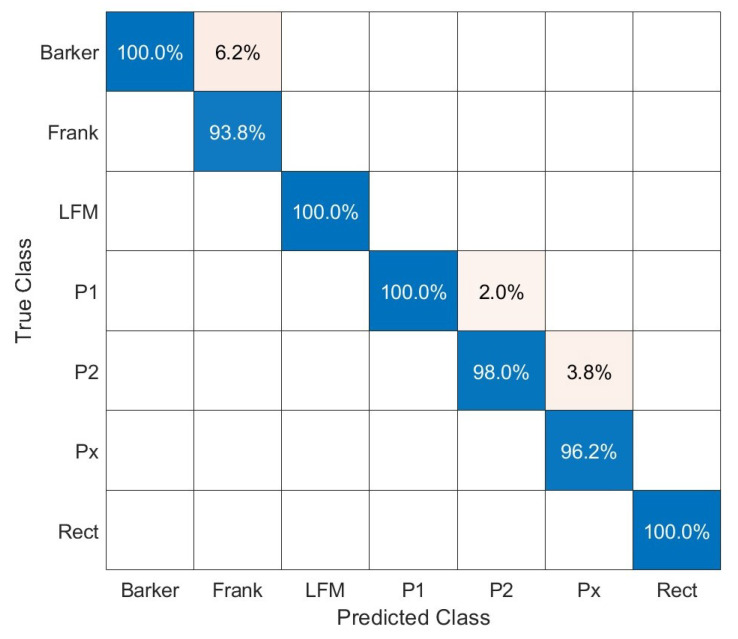
Confusion matrix for AWT with Morse wavelet parameters: γ=9, β=27.

The confusion matrix for parameters with the highest total accuracy corresponding to Table 3 is presented in Figure 10. The overall classification accuracy for γ=9, β=27 was 98.2%.

**Figure 11:**
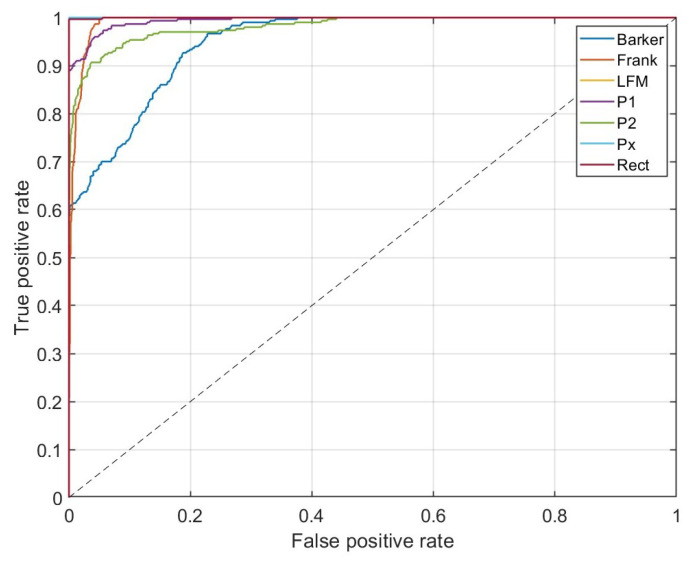
One-versus-all ROC curves for each class for the method with AWT with Morse wavelet parameters: γ=9, β=27.

Additionally, in Figure 11, receiver operating characteristic (ROC) curves for the method with AWT applied (with Morse wavelet parameters: γ=9, β=27) are presented.

The shape of the one-versus-all ROC curve for Barker confirms that their classifier for high-value true positives gives a significant level of false positives, which corresponds to the confusion matrix presented in Figure 10. In the case of Frank, P1, and P2 signals, the one-versus-all ROC curves indicate slightly better performance.

For comparative purposes, the classification process was carried out with SPWVD as TFI and with the Kaiser window with the same parameter values for the smoothing window in frequency and time: window length L = 101 and shape factor N with the selected values. The classification accuracy obtained for each value of N is presented in Table 6.

**Figure 12:**
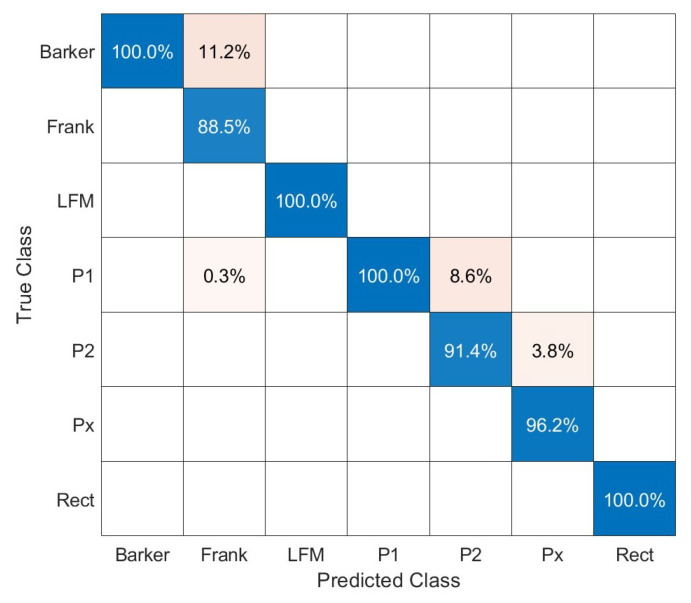
Confusion matrix for WVD with Kaiser window parameters *L* = 101, α=1.

A similar correctness of the classification was obtained for shape factor N = 1, and the confusion matrix is shown in Figure 12. The overall classification accuracy for shape factor N was 96.3%. Lower classification accuracy occurred for P1 and P2 codes, as well as some problems with distinguishing the waveform with the Barker and Frank code. This is a similar situation as for the method with AWT applied.

**Figure 13:**
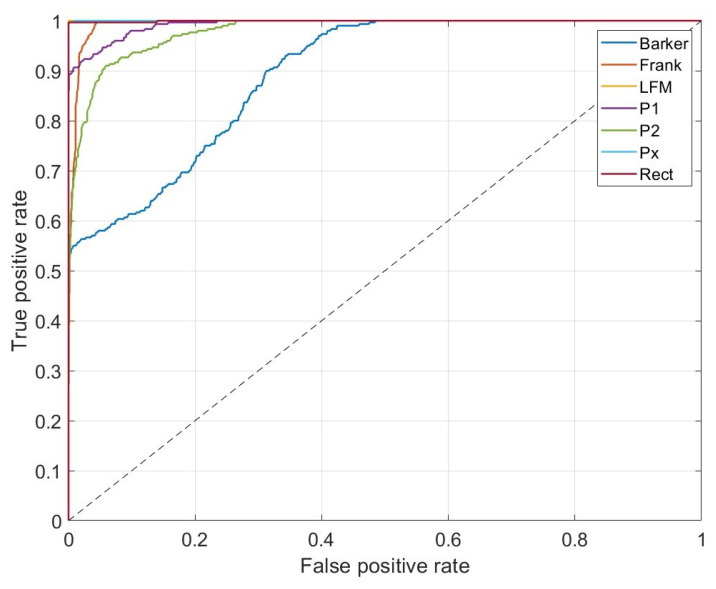
One-versus-all ROC curves for each class for the method with SPWVD with Kaiser window and α=1.

Figure 13 presents ROC curves for the method with SPWVD applied. Similarly, as for the method with AWT applied, there are observable levels of false positives for Barker. In the case of Frank, P1, and P2 signals, the one-versus-all ROC curves indicate slightly worse classification possibilities, almost the same as for the method with AWT applied.

Table 7 and Table 8 list the metrics obtained for both methods, with AWT and SPWVD applied for TFI calculation. Consequently, it is noticeable that the classifier has the ideal ability to distinguish LFM and typical rectangular radar signals (F1-score is equal 1).

## 4. Conclusions

The actual direction of the research works presented in this paper was drawn in one of our previous works [49]. The method of feature extraction based of AWT seems to be very versatile. The results presented in [31] show that using CWT and artificial neural networks (ANN) performs as an effective way to recognize communication signals such as ASK, PSK, FSK, and QAM. The usefulness of the wavelet transform in signal analysis and the more and more commonly used TFI prompted the authors to verify the possibility of using AWT (with parameterized GMW) in combination with a reduced CNN structure (SqueezeNet). Thus, the conducted research works were focused on the influence of the GMW parameters on classification accuracy. The obtained results confirm the satisfactory effectiveness of the proposed algorithm for SNR in the range from −6 to 0 dB. The highest obtained classification accuracy is 98.2% for γ=9 and β=27.

For comparative purposes, the classification process with TFIs based on SPWVD was conducted. It was observed that both algorithms allow for approximate classification accuracy. The advantage of CWT over SPWVD resides in the ability to use a filter bank to improve the efficiency of calculations. On the other hand, for SPWVD, the use of smoothing windows improves the readability of TFI but requires additional time, which was noticed during the simulation tests.

Future work will include the application of a wider range of selected signals with specific parameters such as pulse width, pulse envelope, pulse repetition time, intrapulse modulation, phase coding types, etc. It seems to be reasonable to consider applying adaptive algorithms for wavelet (in CWT/AWT) or smoothing window parameters selection. Another important problem is selecting the type of classifier. Neural network training is a time-consuming process. In the EW systems domain, there is a continuous need for fast methods. Therefore, faster adaptive methods, such as the one presented in [50], even if with relatively lower classification accuracy, are preferable over those that are slower with higher classification rates. Moreover, an interesting alternative worthy of consideration is information fusion methods, referred to in [11], which, apart from the neural-network-based solutions, deliver analytical methods. This may be particularly attractive due to the mentioned speed requirement assessed on the optimizing methods.

Summarizing the presented considerations, the method based on AWT, applied for TFI calculation with the reduced CNN structure (e.g., SqueezeNet), would be more appropriate for hardware implementations then those with STFT or WVD (SPWVD) applied for TFI calculation and with other types of CNN (ResNet, ALexNet, etc.) as classifiers.

Furthermore, by selecting the appropriate wavelet parameters’ values, it is possible to achieve comparable or even better performance test results than those presented in the references.

## Figures and Tables

**Table 1:** Phase-coded waveform parameters.

Code Name	Chip Number
Barker	13
Frank	16
*P*1	16
*P*2	16
*Px*	9

**Table 2:** General simulation parameters.

Parameter Name	Parameter Value
Carrier frequency $F_{c}$	16.7 MHz to 20 MHz
Sample frequency $F_{s}$	100 MHz
Bandwidth (for LFM Waveforms)	from 5 to 6.25 MHz
SNR	from −6 to 0 [dB]
Rician Channel Parameters	PathDelays [0 1.8 3.4]/Fs, AveragePathGains [0–2–10]
Number of pulses in signal	1–2
Number of samples (in observation window)	1024
Number of each type of signal	3000
Number of cycles per phase code (in one Chip)	[7, 8] for Barker, *Px*, [4, 5] for Frank, *P*1, *P*2
Wavelet type	Morse

**Table 3:** The γ and β values taken into account in simulation.

γ	$P^{2}=\gamma \beta$ (β)
3	9 (3), 27 (9), 81 (27), 120 (40)
9	27 (3), 81 (9), 243 (27), 360 (40)
27	81 (3), 243 (9), 729 (27), 1080 (40)

**Table 4:** Additional γ and β values taken into account in simulation.

γ	$P^{2}=\gamma \beta$ (β)
9	81 (9), 99 (11), 117 (13), 135 (15), 153 (17), 171 (19), 189 (21), 207 (23), 225 (25), 243 (27), 261 (29), 279 (31), 297 (33), 315 (35), 333 (37), 351 (39)
27	728 (27), 783 (29), 831 (31), 891 (33), 945 (35), 999 (37), 1053 (39)
50	1500 (30), 1550 (31), 1600 (32), 1650 (33), 1700 (34), 1750 (35), 1800 (36), 1850 (37), 1900 (38), 1900 (39), 1950 (40)

**Table 5:** Total accuracy for parameters corresponding to Table 3.

Parameter Value	β=3	β=9	β=27	β=40
γ=3	83.7%	90.1%	93.6%	96.4%
γ=9	86.6%	94.8%	**98.2%**	97.3%
γ=27	86.1%	91.7%	93.8%	91.9%

**Table 6:** Total accuracy for parameters for selected values of shape factor N.

Shape Factor N	0.5	1	1.5	2	3	4	5
Accuracy [%]	95.9%	96.3%	94.4%	95.6%	94.4%	94.9%	88.9%

**Table 7:** Classification report of the model with AWT applied (Precision, Recall, and F1-Score).

Signal Type	Barker	Frank	LFM	P1	P2	Px	Rect
Precision	0.9412	1	1	0.9800	0.9622	1	1
Recall	1	0.9373	1	1	0.9796	0.9615	1
F1-Score	0.9697	0.9677	1	0.9899	0.9708	0.9804	1

**Table 8:** Classification report of the model with SPWVD applied (Precision, Recall, and F1-Score).

Signal Type	Barker	Frank	LFM	P1	P2	Px	Rect
Precision	0.8992	1	1	0.9186	0.9596	1	1
Recall	1	0.8850	1	1	0.9143	0.9615	1
F1-Score	0.9469	0.9390	1	0.9575	0.9364	0.9804	1

## Data Availability

Not applicable.
